# Promoting Middle School Students’ Science Text Comprehension via Two Self-Generated “Linking” Questioning Methods

**DOI:** 10.3389/fpsyg.2020.595745

**Published:** 2020-10-26

**Authors:** Hava Sason, Tova Michalsky, Zemira Mevarech

**Affiliations:** ^1^Herzog College, Alon Shvut, Israel; ^2^Bar-Ilan University, Ramat Gan, Israel

**Keywords:** scientific literacy, reading strategies, reading comprehension, self-generated questioning, middle school, long-term maintenance, prior knowledge, within-text connections

## Abstract

This quasi-experimental study examined training in two types of reading strategies: self-generated questions either connecting to prior knowledge (Extra-Text) or connecting between the text’s parts (Within-Text). Immediate and long-term effects were assessed on ninth graders’ science text comprehension, versus an untrained control group. The three student groups (*N* = 193) received the same study unit of scientific texts and accompanying tasks, either with/without training in self-generated questioning. PISA-based science literacy assessments (phenomenon identification, scientific explanation, and evidence utilization) were collected at baseline, immediately after intervention, and at 4-month follow-up. Results from both short- and long-term assessments indicated that those learners trained to generate questions about within-text connections reached significantly higher science text comprehension achievements than the other two groups – students trained to generate questions connecting to their prior knowledge and control students who received no support for generating questions. Findings may contribute to the design of support methods and teaching strategies for promoting literacy in general and scientific literacy in particular.

## Introduction

The importance of enhancing science literacy among students of all ages has been emphasized by recent reforms in science education (National Research Council of National Academies, 2011; Organisation for Economic Co-operation and Development [OECD], 2014, 2016, 2017; National Academies of Sciences, Engineering and Medicine, 2016). The Program for International Student Assessment (PISA) defined science literacy as: “The capacity to use scientific knowledge, to identify questions and to draw evidence-based conclusions in order to understand and help make decisions about the natural world and the changes made to it through human activity” (Organisation for Economic Co-operation and Development [OECD], 2003, p. 15). Specifically, these educational reforms encourage the reading of scientific texts, calling on students to “learn how to access scientific information from texts and evaluate and interpret the information they have acquired” (National Research Council of National Academies, 2003, p. 40).

Yet, research has indicated that, when reading scientific texts, students face significant challenges in three major skills for scientific literacy: phenomenon identification, scientific explanation, and evidence utilization (e.g., McNamara, 2017). Namely, many students show substantial difficulties when asked to *identify scientific phenomena* from such texts (Rop, 2003; Michalsky, 2013). Moreover, students often struggle when asked to *give scientific explanations* and to formulate hypotheses based on the texts (Cromley et al., 2010). Finally, when asked to *evaluate and interpret experimental evidence* described in texts, students tend to reject, misinterpret, or ignore data that do not match their existing naïve theories and misconceptions (McNamara, 2017). These three skills’ centrality is also evident from their appearance in international PISA testing of scientific literacy in recent years (Organisation for Economic Co-operation and Development [OECD], 2016, 2017).

These and similar additional research findings on middle-school students (Chin and Osborne, 2010; Fang and Wei, 2010; Okkinga et al., 2018) call for the development of tools for fostering readers’ scientific text comprehension. One highly effective method is for learners to generate self-questions before, during, and/or after reading a passage (e.g., “Do I understand the main idea in this paragraph?” or “What do I already know about this issue?”), aiming to help them monitor and manage their reading comprehension (Gunn, 2008; Kaberman and Dori, 2009; Joseph and Ross, 2018). The process of generating self-addressed questions assists readers in developing higher metacognitive self-regulation concerning the learning process (Moseley et al., 2016). This includes increased focus on critical information; better awareness about texts’ meaning; and improved operations for monitoring understanding, correcting errors, and successfully completing accompanying assignments (Chin and Osborne, 2010; Crabtree et al., 2010; Herscovitz et al., 2012; Wood et al., 2015; Cameron et al., 2017).

Although researchers have begun to investigate students’ self-questions during scientific text reading for their effects on science achievements and scientific literacy (Kaberman and Dori, 2009; Moseley et al., 2016), little empirical attention has been given to the relative effectiveness of different reading strategies underlying such student-generated self-addressed questions. The current study compared two types of self-generated questions that comprise “linking” reading strategies – either connecting to prior knowledge (Extra-Text) or connecting between the text’s parts (Within-Text) – for their immediate and long-term effects on ninth graders’ science text comprehension, versus an untrained control group.

### Bridging (Within-Text) Versus Elaborating (Extra-Text) “Linking” Strategies

Research has demonstrated that successful comprehension of scientific texts relies on readers’ ability to draw links and connections between various sources of information (McNamara, 2007; Kostons and Van Der Werf, 2015). McNamara (2004, 2009, 2017) investigated two essential “linking” strategies for scientific text reading comprehension: bridging inferences and elaboration.

#### Bridging Inferences: Within-Text Links

In the bridging-type strategic process, readers connect between pieces of information that they glean from different parts of the reading task in order to understand the relations between separate sentences, paragraphs, and accompanying visual-graphic representations (McNamara, 2017) like graphs, tables, or diagrams. Making meaning of what has been read derives from the ways in which the various parts and ideas of the science task connect (Kuo and Anderson, 2006). The following presents two examples for bridging activity accompanying a PISA-like text called “Light Cigarettes” for promoting scientific literacy (National Authority for Measurement and Evaluation in Education [RAMA], 2010):

• Bridging multiple parts of a graph: When asked if people who stopped smoking in their 1930s had a similar chance of developing lung cancer as people who never smoked, students need to find the links between different data appearing within a graph that presents information on smokers’ and non-smokers’ ages, quantity of cigarettes smoked, number of years they smoked and type of cigarettes.
• Bridging two parts of text: When asked why the government prohibited labeling such as “lite” for cigarettes in which the amount of tar is low, students need to connect the passage of text describing studies conducted on smokers of “lite” cigarettes and the passage of text describing those cigarettes’ contents.

Often, science tasks do not explicitly pinpoint how the different bits of given written and visual information may complement or clarify one another; readers are expected to infer their causal, temporal, spatial, conceptual, hierarchical, and other interconnections (McNamara, 2007; Barzilai and Eilam, 2018; Jian, 2018). Researchers have asserted that readers’ difficulty in coordinating and connecting (bridging) between different pieces of information that appear within the text often leads to inefficient and decentralized reading (Mason, 2004; Cromley et al., 2010; McNamara, 2017). Research has supported the importance of text-focused reading strategies, which stimulate learners to make connections within the text. Best et al. (2005) suggested that creating inferences between sentences and ideas in science texts can fill perceptual gaps between the learner’s prior knowledge and the new knowledge and help students compete/deal with the level of difficulty of the texts in science books.

O’Reilly et al. (2002) and O’Reilly and McNamara (2007) added that skills for linking parts within a text can help learners to locate comprehension errors while reading the text and to correct them. This builds a system of judgment and control, which allows learners to assess the quality of their learning processes and outputs. Thus, within-text linkages help learners apply meta-comprehension skills that optimize accuracy and that help track progress toward learning goals. To be noted, the inferences made by students in O’Reilly and McNamara’s (2007) studies resulted from instructions given by the teacher to perform such linking activities, and not via students’ own self-questioning.

#### Elaboration: Extra-Text Linkages With Prior Knowledge

In the elaboration-type strategic process, readers link the current text to related knowledge that they already possess. For instance, readers of a text on heart disease need to connect “Coronary artery disease occurs when the arteries become narrowed and hardened” to their previous knowledge that the heart muscle receives blood from the arteries. In addition, readers can also logically apply prior general knowledge to deduce that narrowed arteries would decrease blood flow to the heart muscle, which would cause a lack of oxygen supply that could potentially result in a heart attack. Another example for elaboration activity accompanies a PISA-like text called “Marching and Drinking” for promoting scientific literacy (National Authority for Measurement and Evaluation in Education [RAMA], 2012). In this case, the text referred to the sensation of warmth in the body that occurs when drinking alcohol. To understand the biological processes, readers need to connect to prior knowledge that blood vessels dilate while drinking alcohol, resulting in blood flow at a lower pressure, which causes the body to lose heat and cool down (Organisation for Economic Co-operation and Development [OECD], 2009b).

Previous studies have found that when students connect their prior knowledge to science text reading, their reading comprehension achievements improve (Kendeou and Van Den Broek, 2007; Moos and Azevedo, 2008; Kaberman and Dori, 2009; Ozuru et al., 2009; Moseley et al., 2016; Joseph and Ross, 2018). Kendeou and Van Den Broek (2007) argued that readers cannot be expected to understand the text if they lack the ability to link the new information with their prior knowledge. According to Kendeou and Van den Broek (2007), the previous knowledge that readers bring with them to the text is a tool that allows them to understand the meaning of the words, sentences, and ideas found in the text. Cromley and Azevedo (2007) even explained that the process of searching for a text’s meaning and understanding its main message is defined as a process of building logical connections and completing missing information that relies on the help of the reader’s prior knowledge. Prompts to construct connections between previous and new knowledge were incorporated into Michalsky’s (2013) IMPROVE self-regulation method, using externally generated (rather than self-generated) self-questions such as “What are the similarities/differences between the science text that I am currently reading and the texts that I have read in the past, and why?”

### The Current Study

To examine the effectiveness of two different types of self-generated linking self-questions, as reading strategies for promoting middle-school science readers’ scientific literacy, the present quasi-experiment aimed to compare reading comprehension growth among three groups of students receiving the same study unit of ninth-grade biology texts and tasks. While engaging in this study unit, the Bridging (Within-Text) group underwent training to create self-questions that link between different parts within each task (within the text and between the text and its accompanying visual-graphic representations), whereas the Elaboration (Extra-Text) group was trained to create self-questions linking the current text and accompanying representations to readers’ prior knowledge. The control group did not receive any training to generate self-questions or to focus on linkages within the text or with prior knowledge. Otherwise the control group’s training resembled that of the experimental groups, based on general reading strategies (e.g., highlight unclear terms, reread a paragraph when you don’t understand, etc.) according to the literacy standards of Israel’s Ministry of Education (Pedagogical Secretariat, State of Israel, 2009).

The dependent variable was scientific literacy on biology texts, comprising three skills: (a) identifying scientific phenomena, (b) generating scientific explanations, and (c) utilizing scientific evidence. Beyond collecting assessments of scientific literacy before initiating the 12-week study unit (baseline) and immediately after the unit’s completion (to evaluate short-term effects), follow-up on long-term effects was conducted four months later at the end of the school year. Follow-up aimed to assess the possible lasting effects of the two learning approaches even after the fading of the self-questioning training, while students continued in their natural untrained science lesson environment (Crabtree et al., 2010; see Puntambekar and Hubscher, 2005 for a detailed review on the importance of assessing fading effects in experimental studies). Ninth graders were selected in line with Organisation for Economic Co-operation and Development [OECD] (2006, 2014, 2017) expectations for students of this age to possess these scientific literacy skills, as reflected on PISA tests (Organisation for Economic Co-operation and Development [OECD], 2003, 2006, 2016) conducted internationally in ninth grade. As far as we know, no previous studies have compared students’ attempts to create elaboration linking questions versus their attempts to create bridging linking questions as a means for promoting scientific literacy and specifically for promoting success in solving international PISA tasks in middle-school science learning.

Despite the paucity of research on self-generated linking self-questions, based on findings regarding the beneficial effects of implementing externally generated self-questions into scientific text reading tasks (e.g., Greene et al., 2010; Kostons and Van Der Werf, 2015), we predicted that the students in the two experimental groups (Within-Text and Extra-Text) would outperform the control group on all scientific literacy measures after training. Regarding the two experimental groups’ comparison, in line with previous research on the importance of prior knowledge for reading comprehension of scientific texts (e.g., Gunn, 2008; Kaberman and Dori, 2009; Berkeley et al., 2011; Moseley et al., 2016; Joseph and Ross, 2018), the Extra-Text (Elaboration) group was expected to achieve higher scientific literacy results than the Within-Text (Bridging) group.

## Materials and Methods

### Participants

Participants were 193 ninth-grade students, 89 boys and 104 girls, with a mean age of 15 years (*SD* = 0.64) attending nine classrooms. The middle schools were similar on the following parameters: middle-class socioeconomic status as defined by the Israel Ministry of Education (Central Bureau of Statistics, 2006), and students’ pretest science achievement levels. The five middle-school teachers who were involved in the study (3 female, 2 male; mean age: 33 years, *SD* = 0.82) all held an academic degree in science, were certified for teaching science in middle and high school, and had more than 7 years of experience in science teaching.

Prior to the beginning of the study, the five teachers who taught these nine classrooms were randomly assigned (from the science teachers in the selected schools) to the three research groups, with three classes per group. Thus, two teachers and 57 students were assigned to the Extra-Text group, two teachers and 61 students were assigned to the Within-Text group, and one teacher and 75 students were assigned to the control group. Table 1 presents the distribution of classes, teachers, and students by study group. The 193 participants in this study were those students who completed all pretest, posttest, and follow-up assessments, out of the total number of students in the nine classrooms (*N* = 267).

**TABLE 1:** Sample (*N* = 193) distribution into study groups.

School	Students’ *n*	Teacher	Group			Total groups per school
			
			Extra-Text	Within-Text	Control	
1	59	a		1		3
		b	2			
2	39	c		2		2
3	20	d	1			1
4	51	e			2	2
5	24	e			1	1

### The Intervention

As seen in Table 2, for all three groups, the 12-week study unit (Lessons 3–14) aiming to promote reading comprehension of scientific tasks was designed to correspond with the Israeli national ninth-grade science curriculum (Israel Ministry of Education, 2013) and with the PISA conceptual framework for scientific literacy (Organisation for Economic Co-operation and Development [OECD], 2014, 2017). The five scientific texts and accompanying tasks employed in the study unit for all three groups in the present study (i.e., “Cellular Phone,” “Light Cigarettes,” “Diabetes and Life Habits,” “Height of Brothers,” and “Marching and Drinking” – see Table 2) were suggested by the Israel Ministry of Education, as assignments for promoting scientific literacy (National Authority for Measurement and Evaluation in Education [RAMA], 2012). Throughout the training in all three groups, students and teachers utilized these five PISA-like texts and tasks. Each text comprised a reading passage describing an authentic science-related situation, accompanied by a visual-graphic representation (diagram, graph, or table). Each accompanying task comprised questions of the same type that appear in international PISA tests: open-constructed-response, closed-constructed-response, short-response, multiple-choice items, and complex multiple-choice items.

**TABLE 2:** Summary of research design.

Lesson	Group	Element	Description	References
1–2 (Oct.)	All	*Pretests* for scientific literacy skills	Students complete 8 PISA tasks measuring baseline scores: “Semmelweis’ Diary” – Items: 1, 2, 3, 4, 5, 6 “Tobacco Smoking” – Items: 1, 3	Organisation for Economic Co-operation and Development [OECD] (2006, 2007, 2009a, 2009b)
3–11 (Oct. – Jan.)	All [NOTE THAT ADDITIONS FOR EXTRA-TEXT AND WITHIN-TEXT GROUPS ARE PRESENTED IN CAPS]	*Research process*	Lessons 3–4: Explanation and Demonstration. Teacher explains the importance of reading texts in general and scientific texts in particular. TEACHER EXPLAINS HOW TO CREATE AND ANSWER SELF-QUESTIONS (PER ASSIGNED EXTRA-TEXT OR WITHIN-TEXT GROUP) TO HELP UNDERSTAND SCIENCE TEXTS. Teacher demonstrates how to read and solve the “Cellular Phone” scientific literacy task using various reading skills (e.g., mark unclear words) according to Ministry of Education Department of Science Teaching guidelines (Pedagogical Secretariat, State of Israel, 2009) WHILE GENERATING SELF-QUESTIONS. Students observe the demonstration and participate in the task solution in the whole class. Lessons 5–6: Training in Pairs. Student pairs read the “Light Cigarettes” text and solve the task WHILE GENERATING AND ANSWERING EITHER EXTRA-TEXT OR WITHIN-TEXT QUESTIONS. Teacher moves among pairs and helps if difficulties arise. Lesson 7: Class Discussion. Teacher and whole class discuss pairs’ solutions to the previous “Light Cigarettes” task, THE SELF-QUESTIONS THAT PAIRS POSED (EXTRA-TEXT OR WITHIN-TEXT), AND HOW THOSE QUESTIONS HELPED THEM SOLVE THE TASK. Lessons 8–9: Explanation and Demonstration. Similar to Lessons 3–4, using “Diabetes and Life Habits” scientific literacy task. Teacher explains and demonstrates again how to read and solve the task using reading skills according to Ministry of Education Department of Science Teaching guidelines, WHILE USING THE SELF-QUESTIONING PROCEDURE TO STRENGTHEN STUDENTS’ TECHNIQUE AND ASSIST IN LOCATING DIFFICULTIES. Improvement from previous training in pairs is examined. Lessons 10–11: Training in Pairs. Similar to Lessons 5–6, using “Height of Brothers” task. Lesson 12: Class Discussion. Similar to Lesson 7, referring to “Height of Brothers” task. Lessons 13–14: Task Solution in Pairs and Class Discussion. Similar to Lessons 5–7, student pairs solve the “Marching and Drinking” task and then the class discusses pairs’ task solutions AND SELF-QUESTIONS. Finally, teacher and class summarize and review the study unit on science text reading and task solution UTILIZING SELF-QUESTIONS.	Israel Ministry of Education (2010), National Authority for Measurement and Evaluation in Education [RAMA], 2010, 2012
15–16 (Feb.)	All	*Posttests* for scientific literacy skills	Students complete 8 PISA tasks measuring short-term effects: “Sunscreen” – Items: 2, 4a, 4b “Cloning” – Items: 1, 2 “Ultrasound” – Items: 2, 3 “Genetically Modified Crops” – Item: 2	Organisation for Economic Co-operation and Development [OECD] (2006, 2007, 2009a, 2009b)
17–18 (June)	All	*Follow-up* on scientific literacy skills	Students complete 8 PISA tasks measuring long-term effects: “Evolution” – Item: 1 “Health Risk” – Item: 1 “Tobacco Smoking” – Item: 3 “Tooth Decay” – Item: 3 “Fit for Drinking” – Items: 3, 4 “Mary Montagu” – Items: 1, 2	Organisation for Economic Co-operation and Development [OECD] (2006, 2007, 2009a, 2009b)

The intervention structure and components derived from cumulative research indicating that for students to succeed in posing high-order self-questions to regulate their reading comprehension, they require preplanned orderly guidance, gradual practice, and supportive encouragement (Moseley et al., 2016). Specifically, as detailed in Table 2, for all three groups, the lessons included four phases: (1) *explanation* of the importance of reading texts in general and scientific texts in particular; (2) repeated *demonstrations* (modeling) of how to read the five aforementioned scientific texts effectively and solve their accompanying scientific literacy tasks, using reading strategies (e.g., draw conclusions, hypothesize, raise diverse options for problem solving, isolate variables, represent information in different ways) that science teachers have been instructed to teach by the Israeli government (Pedagogical Secretariat, State of Israel, 2009); (3) *practice*, in pairs, for solving the tasks accompanying the five given texts, presented on printed worksheets; and (4) class *discussion* of pairs’ solutions to tasks.

In the two experimental groups, additional evidence-based features were incorporated into the learning environment to help students learn to pose their assigned self-questions (Extra-Text or Within-Text), with the aim of promoting students’ comprehension of the biology texts. In these two groups, the *demonstration* phase (Phase 2 above) was supplemented by teachers’ *explanation* about the rationale for their assigned self-questioning method and *demonstration* of externally generated self-questions (e.g., Mevarech and Kramarski, 2003). In the *practice* phase (Phase 3 above), the teachers in these two experimental groups added pairs’ *practice* of self-questioning generation (e.g., Michalsky, 2013). In the *discussion* phase (Phase 4 above), the teachers in these two experimental groups added class-wide *discussion* of self-questions (Mevarech and Kramarski, 2003; Michalsky, 2013).

In each of the two experimental groups (Extra-Text and Within-Text), instructions for posing the assigned self-questioning type were integrated into the students’ printed biology task worksheets throughout the text. Students in these groups were helped by these instructions during practice and discussion. Sample instructions for posing an Extra-Text self-question included: “Write a question that refers to the connection between the ________ [results OR methods OR variables] of the research study that you just read and your prior knowledge about this issue.” Sample instructions for posing a Within-Text self-question included: “Write a question that refers to the connection between the last paragraph that you read and _______ [the graph adjacent to the text OR one of the earlier paragraphs in the text].”

Students in the control group received the same study unit and they read and solved the same biology tasks as the other two groups, but without any training regarding self-questions for within-text or extra-text linkages. To ensure that the instruction methods were properly implemented as designed, all five classrooms were observed by the first author every 2 weeks for all four months of the experiment (5 lessons per week × 8 weeks = 40 observations altogether). Observations were conducted of every second lesson where the two self-generated linking self-questioning methods were implemented, and for one random weekly lesson in the control group. The first author, an expert in reading science texts, science literacy, and the differences between the two instructional conditions, met with each of the five teachers after each observation to give feedback, answer questions, and offer recommendations for improvement if necessary. In general, the teachers adhered well to the training they had received, both regarding the science learning unit and the training on reading scientific texts.

### Teacher Training

To prevent treatment diffusion and compensatory rivalry, teachers underwent separate one-day training according to assigned study group and were masked to the other groups’ study procedures. The two teachers assigned to the Extra-Text group were trained together, the two teachers assigned to the Within-Text group were trained together, and the one teacher assigned to the control group was trained alone. To ensure consistency, the same basic training program to impart the pedagogical content knowledge for the ninth-grade science curriculum (except for the addition of the self-questioning contents) was delivered to all teachers by the same instructor (first author).

Training initially introduced all teachers to the importance of enhancing students’ scientific literacy and to the difficulties encountered in comprehending scientific texts. Next, all teachers received the rationales and techniques for the preplanned orderly guidance, gradual practice, and supportive encouragement (Moseley et al., 2016) that they would be implementing while teaching the 12-week study unit – comprising the explanation, demonstration/modeling, task solution, and class discussion procedures. Finally, all teachers observed the instructor as she modeled the assigned group’s student training in a real ninth-grade classroom with students who did not participate in the study.

For the four teachers assigned to the two intervention groups, the instructor additionally discussed the importance of helping students pose their own linking questions to promote students’ comprehension of scientific texts. The four teachers also observed the instructor as she modeled the student training in the assigned self-questioning type (Extra-Text/Within-Text) in the real ninth-grade classroom. The control group teacher received the relevant pedagogical content knowledge and observed the instructor‘s real-time in-class modeling without self-addressed questions.

### Assessments

As seen in Table 2, students’ scientific literacy was assessed at each of the three intervals (Lessons 1–2 at pretest, Lessons 15–16 at posttest, and Lessons 17–18 at follow-up). The tests at the three intervals were conducted under the same conditions in all groups, in the science classrooms at the school that the students attended, during morning hours, with the class teacher present to supervise independent testing performance. All groups received exactly the same tests, comprising a different set of PISA texts with eight accompanying test items at each interval (Organisation for Economic Co-operation and Development [OECD], 2006, 2007, 2009a,b). For example, at the baseline interval, students received two texts, Semmelweis’ Diary with six test items and Tobacco Smoking with two test items (see Table 2). Each PISA text comprised a reading passage depicting an authentic science-related situation, accompanied by a visual-graphic representation. The eight PISA test items assessed at each interval covered the three main skills of scientific literacy: (a) phenomenon identification, (b) scientific explanation, and (c) evidence utilization.

PISA test items’ comparability across the three intervals was maintained for item type (e.g., closed-constructed-response), item level [e.g., according Bloom’s (1956) taxonomy of cognitive categories: knowing, understanding, evaluation, synthesis], and required literacy skill (e.g., phenomenon identification). For example, Semmelweis’ Diary Item 3 at the pretest (Question 1.2, Organisation for Economic Co-operation and Development [OECD], 2009a) as well as Sunscreens Item 2 at the posttest (Question 8.2, Organisation for Economic Co-operation and Development [OECD], 2009a) and Tobacco Smoking Item 3 at the follow-up (Question 24.3, Organisation for Economic Co-operation and Development [OECD], 2009a) were all comparable multiple-choice closed items examining “phenomenon identification.” Likewise, Semmelweis’ Diary Item 1 at the pretest (Question 1.1, Organisation for Economic Co-operation and Development [OECD], 2009a) as well as Sunscreens Item 3 at the posttest (Question 8.4, Organisation for Economic Co-operation and Development [OECD], 2009a) and Evolution Item 1 at the follow-up (Question 28.1, Organisation for Economic Co-operation and Development [OECD], 2009a) were all comparable open items examining “evidence utilization.”

The scoring procedure followed PISA scoring instructions. For open items, scoring was: 0 for incorrect/missing answer, 1 for partial answer, and 2 for a complete answer. For closed items, scoring was: 0 for incorrect/missing answer and 2 for correct answer. Reliability (Cronbach alpha) was 0.74 for the pretest, 0.67 for the posttest, and 0.71 for the follow-up.

### Ethical Procedures

This study was reviewed and approved by our university’s institutional review board and departmental ethics committee, in accordance with the ethical principles of the American Psychological Association. Parents provided written informed consent for their children to participate in this study, and the ninth graders provided their assent, as required by the Chief Scientist in the Israeli Ministry of Education.

## Results

### Total Scientific Literacy

**FIGURE 1:**
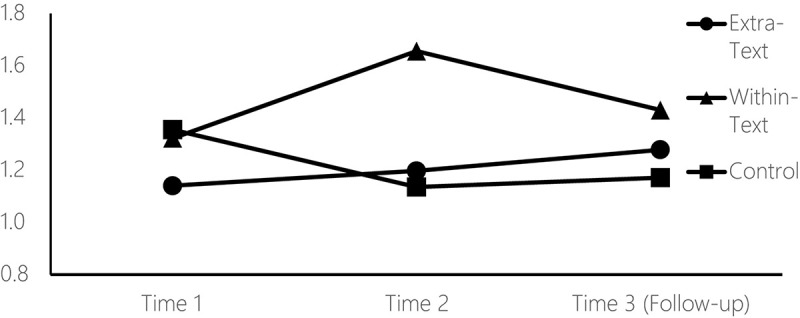
Total scientific literacy at three intervals.

To examine students’ scientific text reading comprehension growth under three instructional methods at the three time intervals, we first examined differences in total scores, using three one-way ANOVAs for each time separately (see Figure 1). No significant difference was found between the groups at the pretest, *F* (2,190) = 2.62, *p* = 0.076, η^2^ = 0.03. However, at the posttest interval (Time 2), significant differences were found, *F* (2,190) = 24.39, *p* < 0.001, η^2^ = 0.20 [*Levene’s test of p-value* = 0.086, meeting the assumption of equality of variance]. *Post hoc* analysis (using Bonferroni) indicated that the Within-Text group significantly outperformed the other two groups after intervention, whereas no significant differences were found at posttest (Time 2) between the Extra-Text and control groups. At the follow-up interval (Time 3) four months after termination of intervention, some of the short-term effects were maintained in the long term, *F* (2,190) = 6.20, *p* = 0.002, η^2^ = 0.06 [*Levene’s test of p-value* = 0.931]. *Post hoc* analysis indicated that the Within-Text group continued to significantly outperform the control group on total scientific literacy scores but did not continue to outperform the Extra-Text group (*p* = 0.164) (see Table 3 for means and standard deviations).

**TABLE 3:** Means and standard deviationsfor scientific literacy scores at three intervals by study group.

Scientific literacy	Time interval		Group		
			
			Extra-Text (*n* = 57)	Within-Text (*n* = 61)	Control (*n* = 75)
**Total**	Pretest	M	1.14	1.32	1.35
		*SD*	0.61	0.44	0.60
	Posttest	*M*	1.20	1.65	1.13
		*SD*	0.46	0.38	0.51
	Follow-up	*M*	1.28	1.38	1.17
		*SD*	0.42	0.45	0.42
**Components:**					
Giving a	Pretest	*M*	1.32	1.42	1.41
scientific		*SD*	0.73	0.65	0.77
explanation	Posttest	*M*	1.34	1.72	1.26
		*SD*	0.62	0.52	0.68
Follow-up		*M*	1.38	1.65	1.13
		*SD*	0.60	0.50	0.56
Utilizing	Pretest	*M*	0.50	0.65	0.77
scientific		*SD*	0.85	0.88	0.88
evidence	Posttest	*M*	0.55	1.41	0.47
		*SD*	0.85	0.86	0.79
Follow-up		*M*	1.11	1.03	1.08
		*SD*	0.57	0.66	0.65
Identifying a	Pretest	*M*	1.40	1.80	1.81
scientific		*SD*	0.92	0.60	0.58
phenomenon	Posttest	*M*	1.55	1.77	1.54
		*SD*	0.83	0.64	0.79
	Follow-up	*M*	1.36	1.52	1.36
		*SD*	0.75	0.68	0.73

*Scores ranged from 0 to 2.*

### Scientific Literacy Components

**FIGURE 2:**
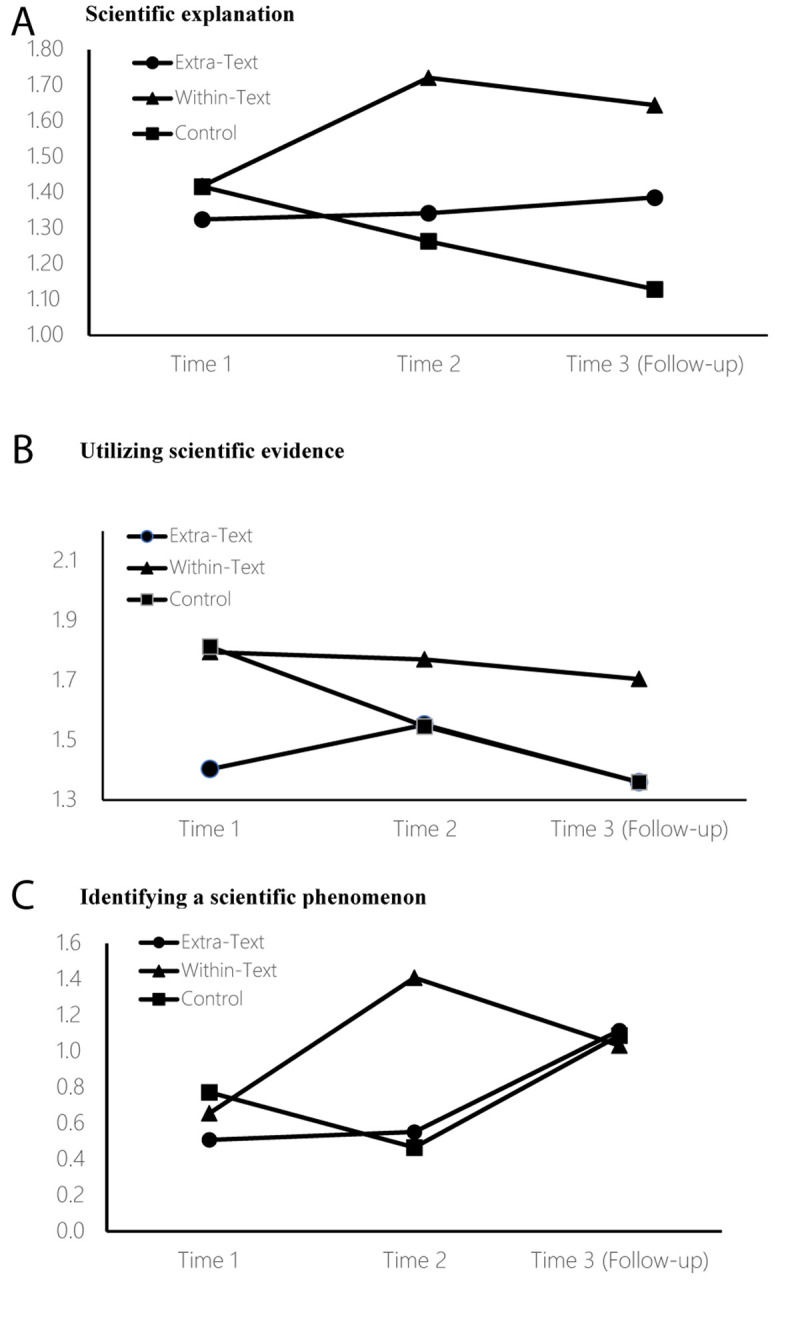
Scientific literacy for the **(A)** scientific explanation, **(B)** evidence utilization, and **(C)** phenomenon identification components.

In the next step of analysis, we examined differences between the three groups in each of the scientific literacy components separately, using a separate one-way MANOVA at each time interval, with the three components as dependent variables. At the pretest (Time 1), a significant difference emerged at the multivariate level, *F* (6,376) = 2.40, *p* = 0.027, η^2^ = 0.04. Univariate tests (see Figure 2A) showed no significant inter-group differences on baseline scientific literacy for either scientific explanation, *F* (2,190) = 0.33, *p* = 0.718, η^2^ = 0.003, or evidence utilization, *F* (2,190) = 1.50, *p* = 0.226, η^2^ = 0.02. For the third component, scientific phenomenon identification, a significant difference was found at the pretest (Time 1), *F* (2,190) = 6.50, *p* = 0.002, η^2^ = 0.06, with the Extra-Text group scoring significantly lower than the control group and the Within-Text group.

At the posttest (Time 2) interval, a significant inter-group differences was found at the multivariate level, *F* (6,376) = 9.60, *p* < 0.001, η^2^ = 0.13 [*Box’s M* = 12.61, *p* = 0.420, meeting the assumption that the variance-covariance matrices were equal across groups]. As seen in Figure 2B, for both of the scientific literacy components that had not shown significant inter-group differences at the pretest (Time 1) interval, a significant difference now emerged in the posttest univariate tests: scientific explanation, *F* (2,190) = 10.08, *p* < 0.000, η^2^ = 0.10, and evidence utilization, *F* (2,190) = 24.96, *p* < 0.000, η^2^ = 0.21. *Post hoc* analysis indicated that, for both of these components, the Within-Text group significantly outperformed the other two groups immediately after intervention. For the scientific phenomenon identification component that had shown significant pretest (Time 1) inter-group differences, no significant differences were found at the posttest (Time 2), *F* (2,190) = 1.76, *p* = 0.175, η^2^ = 0.02.

At the follow-up interval (Time 3) 4 months after termination of intervention, some of the short-term effects were maintained in the long term [multivariate level *F* (6,376) = 6.52, *p* < 0.001, η^2^ = 0.09; *Box’s M* = 20.50, *p* = 0.067]. As seen in Figure 2C, a significant difference was found on the univariate test for the scientific explanation component, *F* (2,190) = 14.51, *p* < 0.000, η^2^ = 0.13. *Post hoc* analysis indicated that, for this measure, the Within-Text group significantly outperformed the other two groups. However, the previous significant inter-group difference found at posttest (Time 2) for the evidence utilization component, favoring the Within-Text group, was not maintained four months later (Time 3), *F* (2,190) = 280.28, *p* = 0.760, η^2^ = 0.003. Regarding scientific phenomenon identification, no significant inter-group differences emerged at the follow-up interval (Time 3), *F* (2,190) = 5.25, *p* = 0.06, η^2^ = 0.05).

## Discussion

This study aimed to promote scientific literacy – now considered a major goal in science education worldwide (National Research Council of National Academies, 2011; Organisation for Economic Co-operation and Development [OECD], 2014, 2016, 2017; National Academies of Sciences, Engineering and Medicine, 2016). The major findings of the current study – examining the effects of ninth graders’ attempts to generate different kinds of self-addressed linking questions while reading scientific texts – were twofold. First, as expected, students’ reading of scientific texts while receiving support for generating either Extra-Text or Within-Text linking self-questions was more effective in developing scientific literacy growth than was reading of scientific texts without such self-questioning support (control group). Second, in contrast to our hypothesis, the students who were trained to generate self-questions about the connections between different parts of the task itself (Within-Text) achieved higher overall scientific literacy than those learners who received training to generate self-questions that connected the text to their prior knowledge (Extra-Text), with some long-term maintenance of these benefits. However, the current outcomes regarding the three main skills comprising scientific literacy (phenomenon identification, scientific explanation, and evidence utilization) may inform the different advantages demonstrated by the two self-questioning strategies in the short and long term.

### The Benefits of Self-Questioning Support During Reading

The advantage found here for both experimental conditions over the control condition coincides with previous studies showing that mere exposure to scientific texts is insufficient, and that explicit instruction is required to train students to self-regulate their own reading (Cromley and Azevedo, 2007). As Hartman (in Schraw, 2001, p. 56) argued:

Teachers should not be satisfied with putting students in situations, which require them to use any strategy they want students to use. Practice isn’t enough. It is also important to provide explicit instruction in **when, why and how** to use the strategy; students need to understand the rationale and effective procedures for the strategy so that they can recognize appropriate contexts for its use, so that they have criteria for evaluating their strategy, and so they can self-regulate its use. [bold is original].

Perhaps the very fact that students in the two experimental groups had to generate self-questions linked to their reading task in itself promoted students’ self-regulated learning processes during reading comprehension, which in turn positively affected their science literacy achievements. Generating self-questioning has the potential to guide students to pay attention to specific aspects of their learning process (Chin and Brown, 2002; Michalsky et al., 2009), thereby helping students to monitor, regulate, and evaluate learning processes. Chin and Brown (2002) found that university students who closely followed self-questions often used these questions as a checklist for reexamining their reading processes and courses of action. Michalsky (2013) concluded that cognitive-metacognitive self-questioning is a self-regulation tool that helps high-school students (10th graders) to shift their attention from procedural thinking to regulation processing, including the construction of sub-goals, the monitoring of learning, and the evaluation of solutions.

### The Benefits of Linking Within the Text Over Linking to Prior Knowledge During Reading

Students in the Within-Text group reached the highest achievements of all groups for total science literacy scores and for two of its three components (utilizing scientific evidence and generating scientific explanations) immediately following the intervention. This advantage of the bridging self-questioning strategy that supported students to make connections within the task – over the self-questioning strategy that supported students to “elaborate” by making connections to prior knowledge and also over the control group’s lack of self-questions – may involve characteristics of these two science skills. Previous studies have pointed out bridging skills (e.g., McNamara, 2004, 2011, 2017; Kuo and Anderson, 2006) and skills for connecting to prior knowledge (e.g., Greene et al., 2010; Kostons and Van Der Werf, 2015) but did not analyze them together. The abilities to locate and use scientific evidence and to offer explanations for scientific occurrences require understanding and reasoning by means of data, facts, and complex multifaceted explications – which often appear in different places in the given text and in its accompanying visual-graphic representations. Thus, practice in posing self-questions to find and understand those connections between the different parts of a science task can assist learners to locate the relevant evidence and put together different pieces of given information to deepen integrative comprehension of complex scientific processes. Kozma et al. (2000) as well as McNamara (2017) argued that the ability to link different parts of the task – such as establishing relationships between paragraphs, sentences in the text, and accompanying graphs, tables, or diagrams – helps the learner to understand the processes and the ideas that appear in the text.

To generate a logical explanatory process, students must find the connections between various ideas and concepts, which was the focus of the self-addressed questioning support received in the Within-Text group. It seems that the ability to formulate scientific explanations relies primarily on various information bits distributed throughout the given task and is probably the least dependent on prior knowledge. Another possibility is that ninth-grade students may have knowledge gaps regarding the scientific topics appearing in these given reading tasks, which may hinder their ability to formulate effective self-questions for activating relevant scientific knowledge (Gunn, 2008; Joseph and Ross, 2018).

Interestingly, regarding the third component of scientific literacy, identifying scientific phenomena, only students in the Extra-Text group demonstrated significant improvement immediately after the intervention. To be noted, these students had shown lower scores than their peers in the other two groups at baseline but caught up after training and even maintained those gains four months later (see below). This finding may be due to the fact that the ability to recognize a scientific process, body, or event requires learners to recall some existing general knowledge on the topic at hand, which was the focus of the self-addressed questioning support received in the Extra-Text group. Additional studies have also mentioned the impact of prior knowledge on understanding scientific texts (e.g., Greene and Azevedo, 2009; Greene et al., 2010; Kostons and Van Der Werf, 2015). In contrast, practicing the generation of connections between the information bits appearing within the science task itself (the Within-Text group) did not appear to offer the extra knowledge needed to identify the wider scientific phenomena being discussed in these biology tasks.

However, the current outcomes indicating that the elaboration (Extra-Text) strategy was not particularly effective overall for promoting science reading comprehension deserve some reconsideration. Different research studies have emphasized that learners’ disciplinary and prior knowledge can critically influence the absorption, processing, understanding, and learning of new information (Greene et al., 2010; Kostons and Van Der Werf, 2015). Yet, perhaps simply asking students to make connections to prior knowledge was an overly general training method because it targeted unfocused non-specific knowledge. This lack of focus may be speculated as having possibly led to students’ cognitive overload, a thought-scattering effect, or repeated searching loops, which may have hindered their ability to find the relevant prior knowledge or to link it appropriately to the given biology task. Researchers have noted the disadvantage of posing questions that lack focus (Davis, 2003; Van den Broek et al., 2006).

### Long-Term Skill Maintenance

In line with recommendations to examine maintenance of intervention gains (Puntambekar and Hubscher, 2005), this study followed up on students’ long-term achievements in scientific literacy four months after the intervention. At the follow-up interval, the achievements of the Within-Text group remained higher than those of the control group both on the total scientific literacy score and on the scientific explanation component. On the scientific explanation component, the achievements of this group were also higher than those of the extra-text group. However, the students in the Within-Text group were unable to maintain the improvement they had achieved immediately after the intervention in their ability to utilize scientific evidence. In this component of scientific literacy, no differences between the groups were found. Perhaps the ability to use scientific evidence may require longer training in order to maintain the gains achieved at the end of the intervention, possibly because this skill is knowledge-specific and therefore relies on memory retention of scientific facts (Kostons and Van Der Werf, 2015; McNamara, 2017).

In the long term, the Extra-Text group no longer showed an advantage over the control group over the control group on the total scientific literacy score or on the other components; however, they were able to maintain the improvement they had achieved immediately after the intervention in their ability to identify scientific phenomena. As mentioned above, this group’s ability to identify scientific phenomena was lower than that of the other groups prior to the training; hence, it can be said that the support they received during intervention to elaborate by seeking relevant prior knowledge outside the text itself was a strategy that continued to significantly help them while reading scientific texts later, after the training supports were withdrawn. Inasmuch as the ability to identify a scientific occurrence always relies on something that students know, it appears that their new ability to ask themselves questions about their own prior knowledge helped them to reach these higher achievements in line with many studies highlighting the importance of prior knowledge (e.g., Kostons and Van Der Werf, 2015; Willis et al., 2019).

### Practical Implications, Future Research, and Limitations

The present findings suggest practical implications for scientific literacy growth programs targeting middle-school students. The Organisation for Economic Co-operation and Development [OECD] (2017) underscored the challenge facing science educators to develop pedagogical models that engage students in authentic, deep forms of inquiry, which promote scientific literacy and thinking as well as metacognition skills and behaviors while reading science texts (McNamara, 2007; Kostons and Van Der Werf, 2015). The current outcomes imply that such programs for middle-school students should focus on the two key elements found here to influence students’ scientific literacy growth: empowering students’ elaboration and bridging types of self-generated linking questions.

The findings of the present study can make a theoretical contribution to the extant research on different types of metacognitive reading strategies and their impact on literacy in general and scientific literacy in particular. Previous studies have pointed to the importance of creating self-questions (Kaberman and Dori, 2009; Moseley et al., 2016; Joseph and Ross, 2018) as well as the importance of making different connections while reading scientific texts (McNamara, 2017). This study combines these two strategies and highlights the unique value of creating self-directed linking questions of different types (extra-text, within-text) for scientific literacy and of its components: (a) phenomenon identification, (b) scientific explanation, and (c) evidence utilization.

Importantly, the current training program and assessments derived directly from the Organisation for Economic Co-operation and Development [OECD] (2017) model for international PISA scientific literacy testing; therefore, this study contributes explicitly to the understanding of which skills can help promote each of the globally recommended literacy components. For example, middle-school students’ ability to utilize scientific evidence and generate scientific explanations are mainly assisted by self-creation of questions that link parts within the text and task, whereas the ability to identify scientific phenomena is mainly influenced by creating self-directed questions that help the student make links outside the text, to prior knowledge. These findings offer practical implications for implementing methods based on metacognitive strategies (Herscovitz et al., 2012) to help students understand scientific texts and even to achieve higher scores on international tests. Hence, this research is also extremely important in terms of its applied contribution and can highlight the need for teacher intervention through different reading strategies and especially through asking different types of linking questions that lead the student to understand scientific texts and success in using different scientific literacy skills.

Another contribution of this study is its follow-up on the effects of metacognitive intervention in the long term, months after training has been terminated. Previous studies (e.g., O’Day and Smith, 2016) have underscored the difficulty in maintaining outcomes over time from interventions that engage students in different reading strategies. The current study likewise found that some gains did not remain, but, in some situations, they were maintained. Future research should continue to delve into possible factors promoting maintenance of scientific reading strategies.

Although implementation of the self-generated linking questions model in middle-school classrooms rendered beneficial effects on students’ scientific literacy, several additional questions remain, both at the theoretical and practical levels. First, it would be interesting in future research to assess this two-approach model for literacy in other content domains like mathematics, chemistry, and physics. Second, recently, the National Research Council of National Academies (2011) published a new framework (*A Framework for K-12 Science Education: Practices, Crosscutting Concepts, and Core Ideas*) that explicitly underscores the need for science teaching as a practice. Practice in this context refers to a way of learning from doing and not (just) from reading and talking science. It would be interesting for future researchers to go beyond mere reading of scientific texts to assess the effects of self-generated linking questions on active science learning through doing, as recommended by these new calls for reform.

As mentioned, a strength of this study is its examination of the intervention’s effectiveness using international PISA tests, which have high validity and reliability (Organisation for Economic Co-operation and Development [OECD], 2006, 2007, 2009a,b). However, it is worthwhile in the future to complement quantitative PISA-based assessments with qualitative methods such as student interviews to shed light on the two intervention groups’ learning experiences as related to the differences in their assessment outcomes. In addition, future research using qualitative methods may compare how text difficulty, domain familiarity, and prior knowledge may affect the way students utilize self-generated linking question instruction as provided while reading scientific texts. Furthermore, to comprehensively scrutinize the issues at hand, researchers would do well to extend investigation to younger students, examine gender differences, and determine teachers’ own skills for self-generating linking questions as playing a possible role in their ability to develop these capabilities among their students (Willis et al., 2019).

## Data Availability Statement

The original contributions presented in the study are included in the article/supplementary material, further inquiries can be directed to the corresponding author.

## Ethics Statement

The studies involving human participants were reviewed and approved by the research reported in this study involving human participants was approved by the Research Ethics Board at Bar-Ilan University. According the ethical standards of the institutional and/or national research committee and with the 1964 Helsinki Declaration and its later amendments or comparable ethical standards. Written informed consent to participate in this study was provided by the participants’ legal guardian/next of kin.

## Author Contributions

TM performed the research design, developed the coding schemes, and supervised the study. HS helped with the data collection and the coding. All authors contributed to the article and approved the submitted version.

## Conflict of Interest

The authors declare that the research was conducted in the absence of any commercial or financial relationships that could be construed as a potential conflict of interest.
